# Evaluation of Functional Features of Lignocellulosic Particle Composites Containing Biopolymer Binders

**DOI:** 10.3390/ma14247718

**Published:** 2021-12-14

**Authors:** Aneta Gumowska, Eduardo Robles, Grzegorz Kowaluk

**Affiliations:** 1 Institute of Wood Sciences and Furniture, Warsaw University of Life Sciences—SGGW, Nowoursynowska St. 159, 02-776 Warsaw, Poland; aneta_gumowska@sggw.edu.pl; 2 University of Pau and the Adour Region, E2S UPPA, CNRS, Institute of Analytical and Physicochemical Sciences for the Environment and Materials (IPREM-UMR 5254), 403 Rue de Saint Pierre, 40004 Mont de Marsan, France; eduardo.robles@univ-pau.fr

**Keywords:** wood, particleboard, binder, mechanical properties, polylactic acid (PLA), polycaprolactone (PCL)

## Abstract

In this research, the assessment of the impact of natural biopolymer binders on selected mechanical and physical properties of lignocellulosic composites manufactured with different resination (12%, 15%, 20%). Different mechanical and physical properties were determined: modulus of rupture, modulus of elasticity, internal bonding strength, thickness swelling, water absorption, contact angle, and density profile. Moreover, thermal properties such as thermogravimetric analysis and differential scanning calorimetry were studied for the polymers. The results showed significant improvement of characterized features of the composites produced using biopolymers. However, the rise of the properties was visible when the binder content raised from 12% to 15%. Further increase of biopolymer binder did not imply a considerable change. The most promising biopolymer within the tested ones seems to be polycaprolactone (PCL).

## 1. Introduction

Currently, the wood-based panels industry appreciates products from renewable sources and biological origin and waste materials as alternatives to the currently dominant products of petroleum origin, of which inventory is known to be limited. Research and experiments are conducted in the scientific community to consider environmental and economic problems by increasing renewable resources in producing wood-based materials. Due to their excellent performance, particleboards are the most used alternative materials to solid wood or plywood because they have a considerably lower cost. Commercial particleboards are usually produced with small wood particles (e.g., shavings, strips, sawdust) using amine resins as adhesive and pressed under high temperature and pressure conditions. In countries that are continually growing wood, the primary raw material for particleboard production decreases every year [1]. Due to the depletion of wood resources for the production of wood-based panels, the use of lignocellulosic agricultural byproducts as a replacement, apart from attempts to use recycled wood-based materials [2], has become a promising alternative [3]. The most common byproduct, which is also rich in lignin and cellulose, are lignocellulosic particles from agricultural residues such as sugar cane bagasse [4], oil palm trunk [5], sugar beet pulp [6], tomato stalks [7], rice husk [8], corn stalk [9], maize cobs [10], coconut husk [11], kenaf stem [12], and waste biomass produced after orchard pruning [13]. In the production of commercial wood-based materials (particleboard, medium-density fiberboard, plywood or oriented strand board), synthetic adhesives such as urea-formaldehyde (UF), melamine-urea-formaldehyde (MUF), and phenol-formaldehyde (PF) are usually used due to their low production cost while maintaining excellent adhesive properties, excellent stability and fast curing [14]. However, they have the disadvantage of free formaldehyde emission during production and during the use of final products, which is a severe problem for health and the environment [15]. Since the end of the 20th century, with increased environmental awareness and improved living standards, the control of formaldehyde emissions is becoming an increasingly important issue [16,17]. Environmental regulations force producers of wood-based panels and wood-plastic composites to think about green technologies because an effective way to control, reduce, or even wholly remove free formaldehyde from products is expected. Many researchers trying to solve this problem realistically have started looking for an environmentally friendly alternative that could be used in products without deteriorating their strength properties.

One proposed solution is to replace non-renewable petrochemical materials with natural and renewable source adhesives. Natural binders include, starch and its modifications [18,19,20], chitosan [21], corn starch [17], tannins [22], citric acid [23], natural latex [24], Arabic gum [25], gelatin, casein, and gluten [26]. Polyhydroxyalkanoates are also increasingly used as renewable source adhesives, which include: polylactic acid (PLA), polyhydroxybutyrate (PHB), and polycaprolactone (PCL), as well as are seen as replacements for commonly used polyolefins [27]. Lignocellulosic wood-based composites using biopolymers as natural binders minimize the negative impact on the environment throughout their life cycle, and they reduce the demand for fuels and petroleum products [28]. The elaboration, preparation, and testing of natural adhesives with good adhesive properties remain an industrial challenge. Owodunni et al. (2020) [29] produced particleboards from coconut fibers (*Cocos nucifera*) using citric acid, native potato starch, and glutardialdehyde modified potato starch as a binder. The best mechanical properties were recorded for samples with the 15% citric acid-modified starch adhesive. 

Another proposed solution for replacing non-renewable petrochemical materials is the production of binderless particleboards in which the phenomenon of self-bonding of the particles depends only on the chemical components contained in the lignocellulosic raw material. Only the pressure and high temperature of the press support the bonding process by exerting pressure that compacts the particles to give the board its final density, thickness, and mechanical strength [30]. Components with adhesive properties include, i.a., polysaccharides, and lignin. 

One of the critical production parameters which influence the properties is the press temperature. Hashim et al. (2011) [31] confirmed the improvement of mechanical and physical properties. Modulus of Rupture (MOR) increased with increasing press temperature (from 160 to 200 °C) and Water Absorption (WA), Thickness Swelling (TS) of the binderless particleboards were improved. Nonaka and collaborators studied the influence of the pressing temperature on the mechanical properties of the particleboards of sugar cane bagasse with PLA as a natural binder [32]. The manufactured particleboard pressed at 260 °C reached a MOR at the level of the reference particleboard, and the TS value was 3.7% lower than the reference samples bonded with diphenylmethylene diisocyanate (PMDI) resin. Baskaran and collaborators produced particleboards of three different thicknesses, 5, 10, and 15 mm, with a target density of 800 kg m^−3^ from the oil palm trunk waste using 10% of PLA as a natural adhesive [33]. Significant improvements in mechanical properties (MOR and IB strength) and physical properties (TS and WA) were observed after adding PLA compared to the reference, binderless particleboard. Moreover, the feasibility of bonding solid wood with PLA and PCL has already been confirmed [34].

The current state of the art on using polyesters (e.g., PLA, PCL, PHB) as a binder in wood-based panels technology is limited, particularly with particleboards. As a result, biocomposites are becoming a more exciting and promising alternative to commonly used petrochemical materials. This investigation aimed to assess the impact of biopolymer binders on selected mechanical and physical properties of lignocellulosic composites manufactured with different resination. The scope of the research included the production of single-layer lignocellulosic composite using natural and renewable sources as binders (especially PLA, PCL). In addition, the final composites with biopolymers were compared to reference composites manufactured following the example of industrial technology with commercial adhesive, such as urea-formaldehyde (UF) resin.

## 2. Materials and Methods

### 2.1. Preparation of the Adhesive

In this research for the production of lignocellulosic composite, such as particleboards, four different binders were used: polylactide (PLA), polycaprolactone (PCL) as well as polystyrene (PS), and urea-formaldehyde resin (UF) as a reference binder. The adhesive mass for individual types of binders (PLA, PCL, PS) was produced by dissolving the dry mass of polymers in solvent to achieve the consistency of a thick liquid. Pure, laboratory-purpose PLA (Sigma-Aldrich, product no. 38534) and PCL (Sigma-Aldrich, product no. 704105) in drops with a diameter of 3 mm were used as biopolymers, while polystyrene in the form of recycled styrofoam elements was used as reference thermoplastic binder. The following solvents have been used to achieve liquid state binders: methylene chloride for PLA, toluene for PCL, acetone for PS. All the solutions proportions have been tuned to achieve the viscosity of solutions close to the reference binder. Urea-formaldehyde (UF) industrial resin (Silekol S-123) was used as a binder for references panels. The hardener for UF glue mass was a 10% water solution of ammonium sulfate ((NH_4_)_2_SO_4_) in a weight ratio of 50:15:1.5, respectively: resin: water: hardener.

### 2.2. Materials Characterization

Single-layer panels were produced under laboratory conditions from softwood particles (95% *Pinus sylvestris* L.). The particles used for produced composites were selected from those passing the 2 × 2 mm^2^ mesh and retained on 0.5 × 0.32 mm^2^ mesh. All the particles have been dried to the moisture content (MC) of about 4%. According to the standard method [35], the bulk density measured was 175 kg m^−3^.

### 2.3. Particleboard Manufacturing

All the composites were manufactured with an aimed density of 750 kg m^−3^ and dimensions 320 mm × 320 mm with a nominal thickness of 4 mm. The resination was kept at 12% in all cases, but additionally, for PLA, PCL, and PS bonded composites, a version with 20% resination was manufactured and for PLA and PCL another version with 15%. In the future, they will be referred to as UF12, PLA12, PLA15, PLA20, PCL12, PCL15, PCL20, PS12, PS20. No hydrophobic agents have been added during manufacturing. The binder liquid solution was sprayed by air gun onto the lignocellulosic particles mixed in a laboratory blender. After blending, the resinated particles were stored in the laboratory fume hood for three days to evaporate the solvents. The lignocellulosic composites were pressed under 2.5 MPa unit pressure with a temperature of 180 °C for boards bonded by PLA, PCL, UF, and 220 °C for boards where the PS solution was used as a binder. Additionally, water in the form of a spray (65 g m^−2^) was introduced into both surfaces of each panel before hot pressing to improve the heat transfer into the core of composites. The total pressing time was 5 min. According to the research plan, the produced composites were conditioned in ambient conditions (20 °C; 65% R.H.) within 7 days before being cut.

### 2.4. Physical and Mechanical Properties

The physical and mechanical properties, where applicable, were determined under European Standards. The modulus of rupture (MOR) and modulus of elasticity (MOE) were determined according to EN 310 [36]. Internal bond (IB) was determined according to EN 319 [37]. For the tests, no less than 12 replicates of each sample were used. Thickness swelling (TS) and water absorption (WA) at two-time intervals, i.e., after 2 and 24 h of immersion in water, were investigated according to EN 317 [38]. Surface water absorption (SWA) was done according to EN 382-2 [39], with at least 12 replicates for each sample. Water contact angle measurements were made using the contact angle analyzer PHOENIX 300 (SEO—Surface & Electro-Optics Co., Ltd., Korea). The contact angle was measured for produced composites of various resination ratios and binders and pure (dry/cured) binders (UF100, PLC100, PLA100, PS100). The density profile (DP) of samples was analyzed using a DA-X measuring instrument (GreCon, Alfeld, Germany). The measurement based on direct scanning X-ray densitometry was carried out with a speed of 0.05 mm s^−1^ across the panel thickness with a sampling step of 0.02 mm. Samples were cut into 50 mm × 50 mm nominal dimensions. No less than 4 samples of every composite type were used to test the DP. Thermogravimetric analysis (TGA) was performed on a Q500 (TA Instruments, New Castle, DE, USA) apparatus in the air (40 mL min^−1^) in the temperature range 50–600 °C at the heating rate of 10 °C min^−1^. Samples of 6–9 mg were tested in two repetitions. Differential Scanning Calorimetry (DSC) tests were conducted using the DSC Q20 Instrument (TA Instruments, New Castle, DE, USA). The tests were carried out at a heating rate of 10 °C min^−1^ under an inert gas (nitrogen) atmosphere with a gas flow of 50 mL min^−1^. Samples of 5 mg were tested in two repetitions. Scanning electron microscopy (SEM) was used to determine the surface morphology of the manufactured composites. For this purpose, a Quanta 200 (FEI, Hillsboro, OR, USA) scanning electron microscope was utilized.

### 2.5. Statistical Analysis

Analysis of variance (ANOVA) and t-tests calculations were used to test (α = 0.05) for significant differences between factors and levels, where appropriate, using IBM SPSS statistic base (IBM, SPSS 20, Armonk, NY, USA). A comparison of the means was performed when the ANOVA indicated a significant difference by employing the Duncan test.

## 3. Results and Discussion

**Figure 1:**
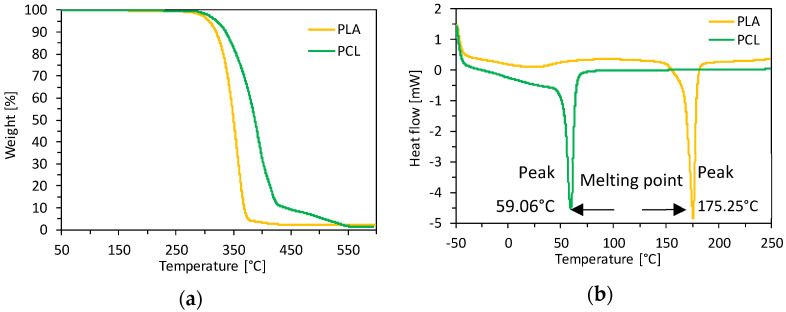
PLA and PCL (**a**) thermogravimetric analysis (TGA), and (**b**) differential scanning calorimetry (DSC).

The TGA and DSC analysis are presented in Figure 1. It is essential to determine the thermal properties to provide the suitability of PLA and PCL for various applications that require integrity at high temperatures, such as particleboard production. Thanks to TGA analysis, the temperature limits (temperature of biopolymers degradation) were recorded, which should not be exceeded during further research with these materials. The data presented in Table 1 displays the thermal stability established for 50% and 80% weight loss of the samples. The higher temperature obtained for PCL is related to higher thermal resistance. According to the literature data on UF resin, the thermal resistance drops significantly in the temperature range of 200–250 [40]. They confirmed that exceeding the temperature of 200 causes the formaldehyde relived from the dimethylene ether groups, which breaks the polymer chain. Thermogravimetric analysis (TGA) for pure PS was conducted by [41]. They confirmed that weight loss reaches 50% at 426.7 °C and 75% weight loss at 437.1 °C. Taking into account the literature data of the UF, PS resin and the obtained results for PLA and PCL, it can be concluded that the lowest thermal stability is achieved by the industrial resin, while PS and PCL weight loss reaches 80% at over 400 °C. DSC curves showed an endothermic melting process at 59.06 °C and 175.25 °C for PCL and PLA, respectively. Typically, PLA has a melting temperature (Tm) in the range of 150–180 °C [42,43]. The characteristic low melting point for PCL was also confirmed [44]. In the wood-based composites production process, the pressing temperature depends on the binder used in the composites. PLA and PCL biopolymer can melt at a pressing temperature of 180 °C and blend well within softwood particles.

**Figure 2:**
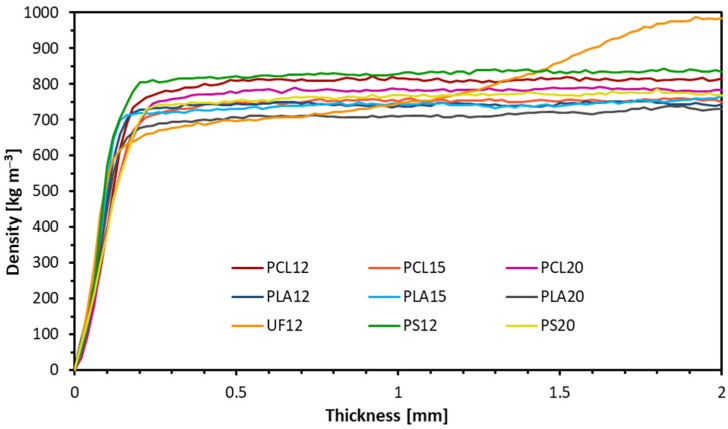
Density profiles of manufactured lignocellulosic composites.

The density profile was characterized, and the results are shown in Figure 2; the average densities of all samples are ~730 kg m^−3^. Since the obtained density profiles for individual samples were symmetrical to the middle of the thickness of the composites, the graph shows the density profiles to their axis of symmetry to facilitate the analysis. When analyzing the results, no change in the density profiles for all composites with polymers was noticed. The presented profiles are flat over the entire cross-section, regardless of the thermoplastic biopolymer binders or the share of resination. No significant connections between the amount of binder used and density profile were found. The typical density profile in these wood-based panels is a characteristic density profile where significant differences between surface and core layers are visible [13]. The density profile of the composite made with industrial resin UF shows a characteristic increase in the density precisely in the middle of the sample thickness. The increase in the density for composite with UF resin might be because UF is a thermosetting resin, unlike the used thermoplastic polymers. The effect of the pressing parameters (pressure and temperature) is transmitted to the middle of the boards resulting in a complete hardening of the resin. However, in the case of composites with thermoplastic polymers, it can be assumed that after extracting the composites from the press, no hardening was obtained. The lack of pressure exerted by the press shelves allowed the density to be evenly distributed over the entire section while the composites were still hot. After cooling down, they reached a temperature lower than the melting point of the biopolymer, keeping the obtained result. The second factor that could explain the unique profile for UF12 from the remaining binders is the amount of water. In the production of adhesive masses, only in the case of UF resin, an amount of 23% (*w/w*; regarding solid resin content) of water was included (technologically, by resin provider) in the adhesive mass. Wong et al. (2000) [45] produced fiberboard with a flat, homogeneous, and typical U-shaped (conventional) density profile, where one of the processing parameters was the manipulation of moisture content distribution. Suzuki and Miyamoto (1998) [46] determined the effect of resin content (RC) on the density profile of homogeneous particleboard with various RC. They confirmed that low RC and low panels’ moisture content were why a higher density surface layer was not formed in this kind of board during hot pressing. The density of surface layers became higher in the core when the RC of the particleboard increased.

**Figure 3:**
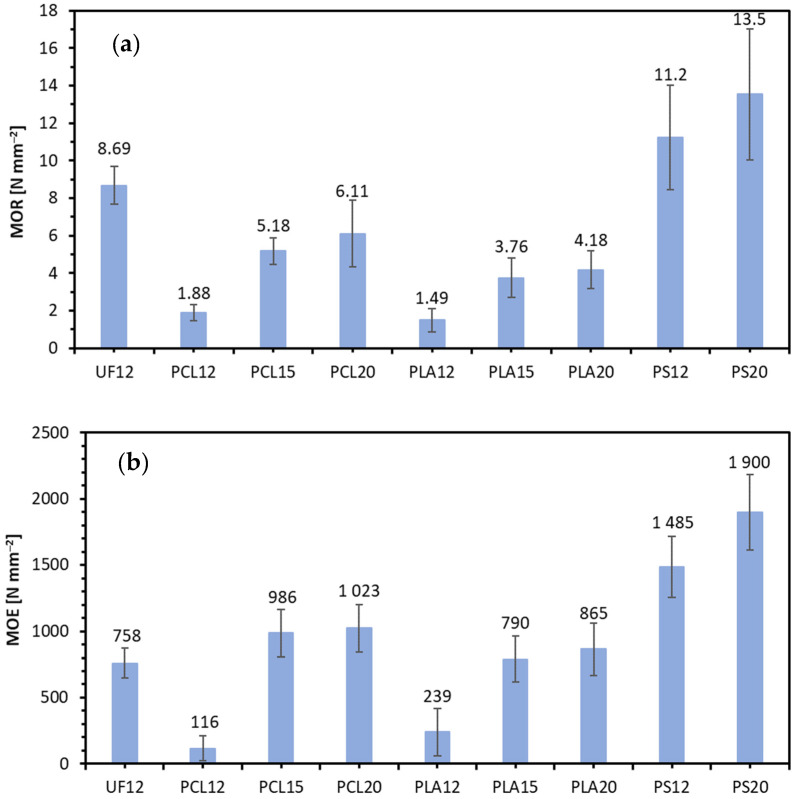
(**a**) Modulus of rupture (MOR) and (**b**) modulus of elasticity (MOE) of tested lignocellulosic composites.

The modulus of rupture (MOR) and modulus of elasticity (MOE) under the three-point bending stress of the investigated panels are presented in Figure 3. As it can be seen, the highest value of MOR (13.5 N mm^−2^) was found for PS20, while the lowest (1.5 N mm^−2^) was for PLA12. The reference composite, UF12, has a MOR value at the level of 8.7 N m^−2^. In the case of MOE, the highest average value was achieved for PS20 (1900 N mm^−2^) and MOR. The lowest was for PCL12 (116 N mm^−2^). For PLA12 and PCL12, the average MOE values are significantly different from the remaining average values. There are no statistically significant differences between average MOR values of samples UF12, PCL15, PCL20, PLA15, PLA20. There are also no such differences between both PS composites. It can be seen that for each of the polymers used, the relationship is directly proportional; along with the increase in the resination in the lignocellulosic composite, the average MOR and MOE value also increases. The composites’ 12% resination of biopolymers gives the lowest average values of bending strength and modulus, but just an increase to 15% translates into results comparable to the results for reference composite with UF resin. Regardless of the polymer used as a binder, the highest average strength values are for every panel with 20% resination. The density profiles of composites, except reference (UF12), are flat and uniform along with the board thickness, any change in the bending properties could be attributed to the difference in the composite mean density, as long as the sample does not experience shear failure during the static bending test [47]. According to statistical analysis, there are no significant differences between the average values of the MOR results for PCL15–20, PLA15–20, and between UF and PS binders. When referring to the achieved MOR and MOE results to standard requirements [48] for panels intended to interior fitments (including furniture–P2 type), it should be said that since minimum MOR for panels of thickness 4 mm and above is 12 N mm^−2^, the only panel meeting this requirement is PS20. The same is for MOE, where standard minimum requirement [48] is 1950 N mm^−2^.

**Figure 4:**
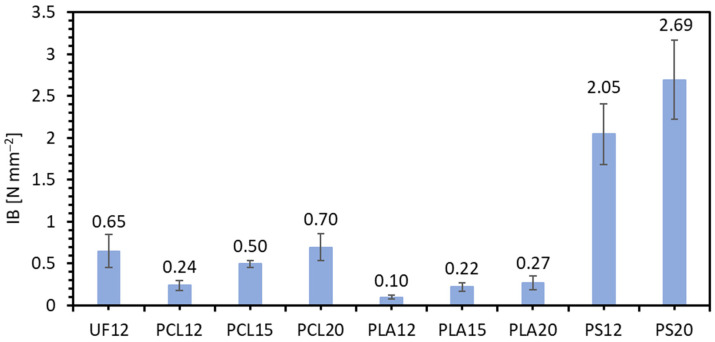
Internal bond (IB) of tested lignocellulosic composites.

**Figure 5:**
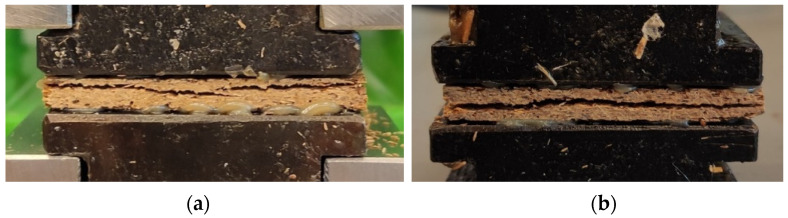
Two representative forms of damage after the IB test (**a**) near-surface zone (**b**) in the core layer.

The obtained internal bonding (IB) tests are presented in Figure 4. The results show that the highest average value of IB was that of PS20 (2.69 N mm^−2^) and the lowest value for PLA12 (0.10 N mm^−2^). As for the MOR and MOE results, the average IB value increases with increasing resination. The predominant forms of damage of the samples resulting after the IB test are summarized in Figure 5. Two representative forms of damage were distinguished during the analysis of all damage images for each sample of lignocellulosic composites. The first group of damage in the near-surface zone includes samples with an average IB below 0.5 N mm^−2^. The second group includes samples in which the destruction took place in the middle of the composite thickness. The second form of destruction was obtained by UF12 (0.65 N mm^−2^), PCL20 was 0.70 N mm^−2^—8% more than industrial resin, and PS achieved a significantly higher average value in both resination. For PLA, low average IB values were obtained, and the destruction occurred for all of PLA in the subsurface layers, the weakest place in the entire cross-section of the sample. Since the mechanical strength of lignocellulosic composites, as IB is, varies on density, it can be found that the face layers density of PLA composites (Figure 2) is low and wide. This effect can be minimized by mechanical correction of the density profile of produced composites by sanding off face layers, as it is made in the case of commercially available particleboards. Aside from PS12, PS20, and other binders among the PCL, only PCL20 have recorded failure in the core layer, thus obtaining the same result as UF12, therefore the second representative form. Destruction in the core layer proves the stronger bonding between the particles in surface layers. Wong and collaborators produced particleboards with homogeneous and conventional (U-shape) density profiles [45]. At equal average density, the MOR and MOE of the conventional particleboards are higher than the homogeneous boards due to the higher density near the faces. At the same time, an increase in the core density results in better IB and inter-particle contact, which allows a more effective bonding. The adhesive covers a larger particle surface instead of filling the spaces in-between the particles [47]. According to Baskaran and collaborators [33], the internal bond strength (IB) of oil palm trunk particleboards was significantly influenced by the addition of PLA (10%) and particleboard thickness. In the present work, statistically significantly different results of average IB were noticed between PS12 and PS20 compared to the remaining results. Furthermore, statistically significant differences were obtained between PLA 12 and other PLA samples, similar PCL12, and other PCL samples.

**Figure 6:**
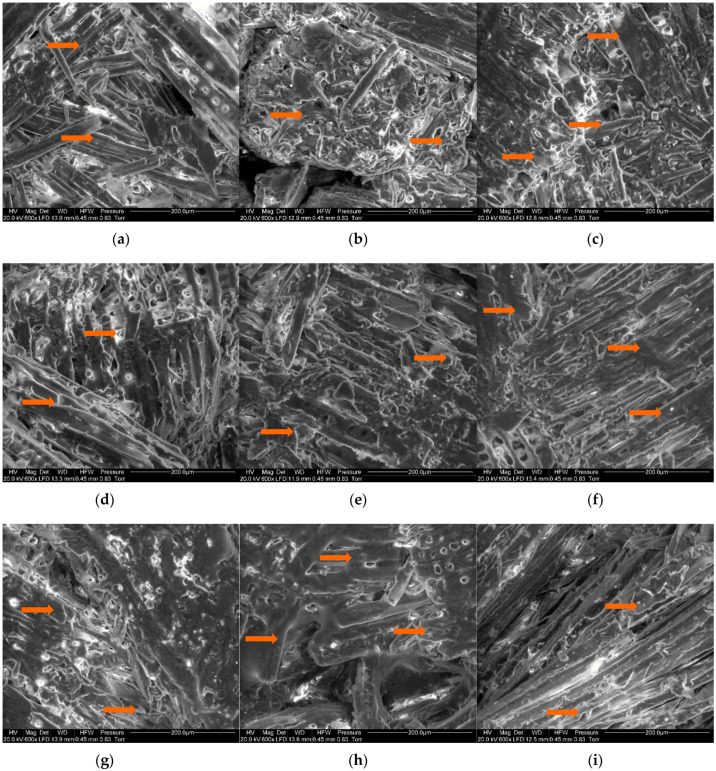
Scanning electron microscope images of lignocellulosic composites with (**a**) PLA12, (**b**) PLA15, (**c**) PLA20, (**d**) PCL12, (**e**) PCL15, (**f**) PCL20, (**g**) PS12, (**h**) PS20, (**i**) UF12; zones covered by binder indicated by arrows.

Scanning electron microscopy (SEM) was applied to observe the surface of lignocellulosic composites with polymers and UF resin directly on a microstructure scale. The photos are presented for each composite and marked with arrows of the distinct areas where clearly can be appreciated how the wooden particles are covered by the binders (Figure 6). There is a visible increase in the amount of binders (different resination) covering the wood fibers, from 12% to 20%, potentially affecting mechanical and physical properties. Additionally, the composite—water interaction (such as TS, WA, and SWA) can be dependent on the tested materials microstructure and binder distribution.

**Figure 7:**
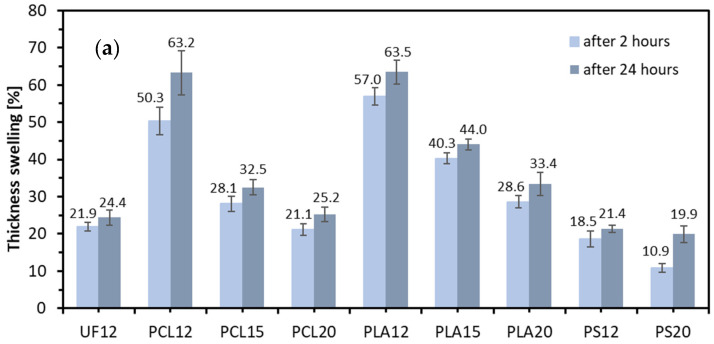

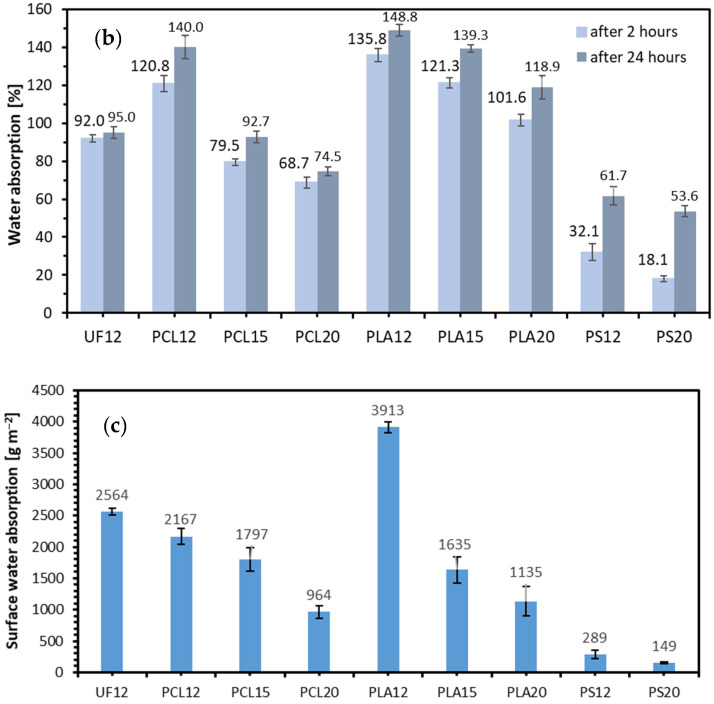
(**a**) Thickness swelling, (**b**) water absorption, and (**c**) surface water absorption of tested lignocellulosic composites.

**Figure 8:**
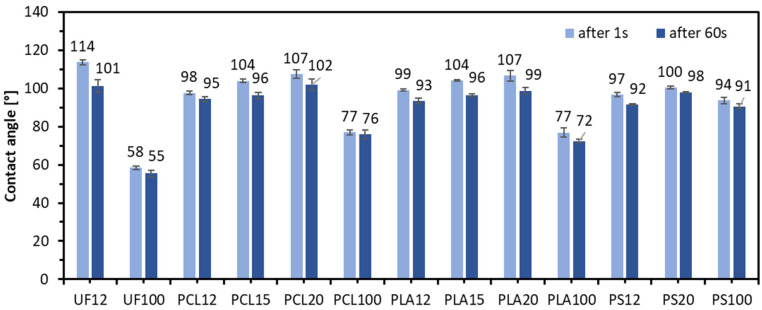
The contact angle of tested lignocellulosic composites and pure binders.

The thickness swelling (TS) and water absorption (WA) after 2 and 24 h average values of lignocellulosic composites are presented in Figure 7. The average TS of the specimens after 2 h of immersion ranged from 10.9% for PS20 to 57.0% for PLA12. After 24 h of immersion, the results were between 19.9% and 63.5%, in the same order as mentioned above. The intensity for TS, calculated as the percentage difference between 2 h TS and 24 h TS, was recorded highest for PLA15 (91.5% of total TS after 2 h), 90% for UF12, while the lowest was for PS20 (54.6%), a paraffin emulsion (or other waxes) is added to the particles during the production of commercially available boards to ensure water resistance under industrial conditions. In the above tests, no hydrophobic agents were used to produce the tested composites. The reference composites use UF resin, intended to be used in dry conditions. It can be appreciated that the TS decreases with the increase in the resination. As with the mechanical properties, it can be seen here that applying a resination level of 15 or 20% gives results with similar average values to composites made with an industrial resin. The 12% of resination of polymers was not enough to obtain results compared to references composites. Referring to the SEM analysis carried out above, it can be confirmed that the increase of resination visibly shows the larger particle surface covered by polymer, which translates into a limited sorption process. WA of the tested composites after 2 and 24 h of immersion in water show a trend consistent with the results of TS. PLA12 had the highest value of WA either after 2 and 24 h, while the lowest obtained PS20. The highest spread between 2 and 24 h, recognized as the lowest intensity of WA, was 29.6% and 35.5% for PS12 and PS20, respectively. The results obtained for TS and WA are confirmed in the conducted surface water absorption (SWA) test (Figure 7). The lowest SWA average value was recorded for PS20 (149 g m^−2^) and the highest for PLA12 (3913 g m^−2^), following the same trend as in TS. The low amount of water uptake by samples bonded by PS can be explained by the high contact angle of this binder (PS100), which means weak water spread on the surface (Figure 8).

Moreover, when analyzing the highest SWA and referring it to 2 h WA, it can be found that these values are strongly connected. The 3913 g of water taken by a 1 m^2^ panel 4 mm thick and of 730 kg m^−3^ density means that the weight of the panel raised by 134%, which means almost the same as when soaked 2 h during WA testing (135.8%). The PLA12 samples after SWA were fully impregnated by water, even if water was acting on samples from one (top) side only. This means that the coverage of the wooden particles by PLA in such a low resination (12%) was very weak (can also be seen in Figure 6a), the structure was highly porous, and the water was easily transferred through the entire structure. When the PLA resination was raised to 15%, the SWA decreased to 1635 g m^−3^, which means over 58% reduction of SWA with 3% rise of resination.

Further resination raise to 20% cause continued SWA reduction of about 12.8 percentage points about PLA15 SWA. What should be pointed, except high SWA of PLA12 samples, the remaining samples of PCL showed very similar SWA as in the case of the same resination of PLA. According to statistical analysis, there are no statistically significant differences between the average values of the SWA results for PCL15 and PLA15, nor PCL20 and PLA20. However, the remaining average values were statistically significantly different.

In Figure 8 were presented results of contact angle for all produced lignocellulosic composites, and wider characteristics were measured for pure polymers and UF resin. This test shows that the sessile drop water contact angle is more hydrophobic for every binder with 20% resination. One of the exceptions is UF12, where a similar average contact angle can be observed regardless of the resination level. It was noted that increasing the resination of polymers decreased the porosity of the composite material. Therefore, as the resination increased for both 1 s and 60 s, the average value of the contact angle was higher. The highest contact angle was recorded for UF12, 114° (1 s) and 101° (60 s), the lowest for pure UF resin, 58° (1 s) and 55° (60 s). It is worth adding that the contact angle decreased after 60 s of water droplets remained on the tested surface. These changes were highest for UF12 (10.9% reduction), PCL15, PCL20, PLA15, PLA20—7.4%, 5.1%, 7.4%, 7.4%, respectively. The smallest changes of contact angle after 60 s were found for pure binders: 5.0% for UF100, 1.5% for PCL100, 5.8% for PLA100, and 3.4% for PS100. The above-mentioned high changes of contact angle on the surfaces of wooden composites can be explained by the activity of the microporous surface of wood particles.

## 4. Conclusions

The conducted research on the application of different ratios of biopolymer binders, such as PCL and PLA, when producing particle structure wood-based composites, allows the formulation of the following conclusions and observations:

1. The density profile of the composites produced using biopolymers is significantly different from this for composites made of commercial UF resin. Biopolymer bonded composites had a homogeneous (flat) density profile, irrespectively of binder amount.
2. Both MOR and MOE significantly rise when the biopolymer binder content rises. The MOR values are about 3 times higher with the resination increase from 12% to 20%. In the case of MOE, it was 880% higher, while PCL content rose from 12% to 20%, and over 360% raise for PLA content increased from 12% to 20%.
3. A specific density profile can influence the low IB of composites bonded with biopolymers. Further attempts to modify the density profiles by sanding can validate this remark.
4. The increase of biopolymer binder significantly influences the TS, WA, and SWA of tested composites. However, the changes are most intensive when the resination raises from 12% to 15%.
5. Regarding achieved results of MOR, MOE, IB, TS, WA, and SWA, it can be concluded that the increase of biopolymer binder content from 15% to 20% seems unjustifiable.
6. The most promising biopolymer within the tested ones seems to be polycaprolactone (PCL).

## Figures and Tables

**Table 1:** Thermal stability estimators for the investigated biopolymer samples.

Tested Materials	Mass Loss	
	50%	80%
	°C	
PLA	349.3	363.3
PCL	387.4	413.8

## Data Availability

The data presented in this study are available on request from the corresponding author.
